# Highly Dispersed Vanadia Anchored on Protonated g-C_3_N_4_ as an Efficient and Selective Catalyst for the Hydroxylation of Benzene into Phenol

**DOI:** 10.3390/molecules27206965

**Published:** 2022-10-17

**Authors:** Juanjuan Liu, Haoyong Yin, Qiulin Nie, Shihui Zou

**Affiliations:** 1 College of Materials & Environmental Engineering, Hangzhou Dianzi University, Hangzhou 310036, China; 2 Key Lab of Applied Chemistry of Zhejiang Province, Department of Chemistry, Zhejiang University, Hangzhou 310027, China

**Keywords:** benzene hydroxylation, vanadia, g-C_3_N_4_, phenol, protonation

## Abstract

The direct hydroxylation of benzene is a green and economical-efficient alternative to the existing cumene process for phenol production. However, the undesired phenol selectivity at high benzene conversion hinders its wide application. Here, we develop a one-pot synthesis of protonated g-C_3_N_4_ supporting vanadia catalysts (V-pg-C_3_N_4_) for the efficient and selective hydroxylation of benzene. Characterizations suggest that protonating g-C_3_N_4_ in diluted HCl can boost the generation of amino groups (NH/NH_2_) without changing the bulk structure. The content of surface amino groups, which determines the dispersion of vanadia, can be easily regulated by the amount of HCl added in the preparation. Increasing the content of surface amino groups benefits the dispersion of vanadia, which eventually leads to improved H_2_O_2_ activation and benzene hydroxylation. The optimal catalyst, V-pg-C_3_N_4_-0.46, achieves 60% benzene conversion and 99.7% phenol selectivity at 60 ^o^C with H_2_O_2_ as the oxidant.

## 1. Introduction

Phenol, one of the most valuable organic intermediates for fine chemical manufacturing [1,2], is produced industrially by a three-step cumene process from benzene [3]. Unfortunately, this process suffers from high energy consumption, relatively low yield, and large amount of acetone as by-products [4,5]. To address these problems, extensive efforts have been devoted to developing an economical efficient and green approach for phenol production. Among various methods, the direct hydroxylation of benzene to phenol using H_2_O_2_ as an oxidant has been recognized as a promising alternative as it can be operated under mild conditions and only produces water as a co-product [4,6]. However, because phenol is more reactive to oxidation than benzene, obtaining high selectivity to phenol at high benzene conversion is difficult [7,8]. To this end, a key topic for the catalytic hydroxylation of benzene is the development of advanced catalysts to achieve high activity and benzene selectivity simultaneously.

Recently, graphitic carbon nitride (g-C_3_N_4_) has emerged as a fascinating material to load active metals for benzene hydroxylation [9,10,11,12]. As an analog of graphite, g-C_3_N_4_ possesses a stacked 2D structure with π-conjugated planar layers, which endow it with high capability in activating aromatic molecules (e.g., benzene) [13]. Meanwhile, the rich uncondensed aliphatic amines, in the form of –NH_2_ and –NH– groups, provide abundant anchor sites for active species [14,15,16]. Because the planar adsorption of benzene on the surface of g-C_3_N_4_ is stronger than the non-planer adsorption of phenol [17], g-C_3_N_4_-supported catalysts usually exhibit good phenol selectivity in benzene hydroxylation, regardless of the supported transition metals (Cu, Fe, V, Co, Ni). The key problem for g-C_3_N_4_-based catalysts then becomes “how to improve the benzene conversion”. A frequently used strategy is to optimize the type and loading amount of metal sites. For example, Ding et al. [4] synthesized a series of metal-doped g-C_3_N_4_ catalysts and found that V-g-C_3_N_4_ was the most efficient one. Under optimal conditions, V-g-C_3_N_4_ can achieve 18.2% benzene conversion and 100% phenol selectivity. Wang et al. [18] optimized the loading of vanadia and found that 8V/g-C_3_N_4_ exhibited superior activity with a benzene conversion of 24.6% and phenol selectivity of 99.2%. Recently, Wang et al. [8] doped cerium into V/g-C_3_N4 catalysts by a simple co-assembly strategy and found that Ce_0.07_/0.07V/g-C_3_N_4_ greatly improved catalytic activity with 33.7% benzene conversion and 95.9% phenol selectivity.

At a fixed loading amount of vanadium, the structure of g-C_3_N_4_ also affects the catalytic performance of V-g-C_3_N_4_. For example, using mesoporous carbon nitride as a support material can increase the surface area of g-C_3_N_4_ and the dispersion of vanadia to improve the benzene conversion [19,20]. Recently, Xu et al. reported that exfoliated and protonated g-C_3_N_4_ can provide more anchor sites for the immobilization of active species [21]. They proposed that the chemical environment (especially its nitrogen species) of g-C_3_N_4_ is critical to the loading of vanadia. Notably, the protonation of g-C_3_N_4_ was usually conducted in strong acidic conditions, which not only change the local chemical environment but also the surface area. To investigate the intrinsic influence of the chemical environment of g-C_3_N_4_ on the vanadia dispersion and the catalytic performance, a synthetic method that can regulate the surface structure of g-C_3_N_4_ without changing the bulk structures is highly desired.

Herein, we develop a one-pot synthesis of V-pg-C_3_N_4_ to regulate the surface amino groups of g-C_3_N_4_ without changing the bulk structure and surface area. Characterizations and catalytic tests suggest that the content of surface amino groups is a decisive factor for the dispersion of vanadia and can quasi-linearly influence the catalytic performance of V-pg-C_3_N_4_ in the direct hydroxylation of benzene to phenol. A maximum of 60% benzene conversion and 99.7% selectivity to phenol can be achieved at 60 ^o^C on V-pg-C_3_N_4_-0.46, which is attributed to the high dispersion of V species stabilized by amino groups, the presence of V^4+^/V^5+^ redox pairs, and the cooperative benzene-activation capability of g-C_3_N_4_. The mass specific activity of V-pg-C_3_N_4_-0.46 is as high as 4.26 and 2.11 g g_cat_^−1^ h^−1^, which exceeds most reported results (Table 1).

## 2. Results and Discussion

### 2.1. Bulk Structures and Surface Structures of V-g-C_3_N_4_ and V-pg-C_3_N_4_

**Figure 1:**
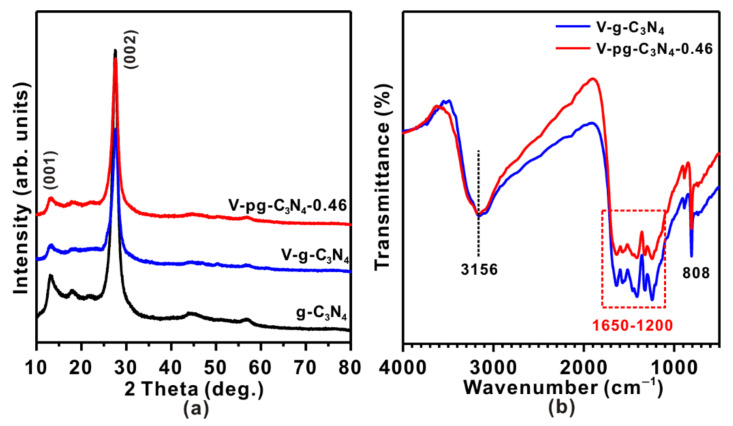
(**a**) X-ray diffraction (XRD) patterns and (**b**) Fourier transform infrared spectroscopy (FT-IR) spectra of as-synthesized samples.

Unlike traditional methods that protonate g-C_3_N_4_ before loading vanadia [21], the one-pot synthesis of V-pg-C_3_N_4_ was conducted in a mixture of HCl, g-C_3_N_4_, and vanadyl acetylacetonate where the protonation of g-C_3_N_4_ and the loading of vanadia took place simultaneously. V-g-C_3_N_4_ was synthesized in the same way as V-pg-C_3_N_4_-0.46 except that no HCl was added. As shown in Figure 1a, the diffraction patterns of V-g-C_3_N_4_ and V-pg-C_3_N_4_-0.46 are very similar to those of g-C_3_N_4_, suggesting that vanadia loading and protonation treatment exert little influence on the bulk structure of C_3_N_4_. Characteristic peaks at 27.5° and 13.6° correspond to (002) and (001) planes of graphitic C_3_N_4_, which are in accordance with the interlayer stacking structure of aromatic systems and the in-plane reflection of the tri-s-triazine motifs, respectively [31]. N_2_ sorption measurements show that g-C_3_N_4_, V-g-C_3_N_4_, and V-pg-C_3_N_4_-0.46 have similar surface areas (ca. 11 m^2^ g^−1^), excluding the exfoliation of bulk g-C_3_N_4_ during the protonation treatment in the one-pot synthesis. No distinct peaks related to vanadia-containing phases are observed in V-g-C_3_N_4_ and V-pg-C_3_N_4_-0.46, likely due to the low mass loading and high dispersion of vanadia species [32].

Consistent with the similar bulk structure, the samples exhibit very similar FT-IR spectra (Figure 1b). The broad bands at ~3156 cm^−1^ can be ascribed to the stretching vibration of uncondensed amino functional groups (such as -NH- and -NH_2_) on the graphitic sheets and the O-H groups of the adsorbed water [33,34]. The characteristic bands at 1200–1650 cm^−1^ correspond to the stretching and rotation vibration of C-N and C=N in heterocycles [35]. The sharp peaks at 808 cm^−1^ are usually treated as the breathing modes of triazine units (C_6_N_7_, the building blocks of the g-C_3_N_4_ structure) [36,37,38]. Notably, though the peak location and shape are similar, the intensity varies, indicating their different local structures. Specifically, the intensity of the bands at 1200–1650 cm^−1^ ascribed to aromatic heterocyclic rings obviously weakens on V-pg-C_3_N_4_ in comparison with that of V-g-C_3_N_4_ while the intensity of the bands at 3156 cm^−1^ is almost identical. These results indicate that the addition of HCl in the preparation would cleave some heterocycles in g-C_3_N_4_. According to the literature [21,39], such cleavage usually promotes the formation of defects sites and terminal N species (e.g., C-NH*_x_*). Considering the similar bulk structures of V-g-C_3_N_4_ and V-pg-C_3_N_4_, the cleavage of heterocycles likely occurred only on the catalyst surface.

**Figure 2:**
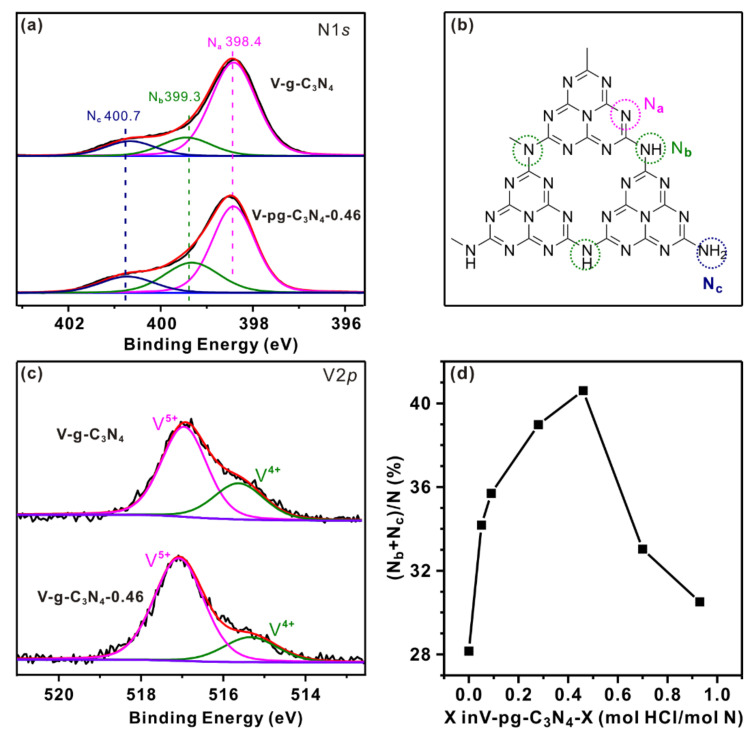
XPS analysis of V-g-C_3_N_4_ and V-pg-C_3_N_4_-0.46 samples. (**a**) N1*s*; (**b**) a possible structure of g-C_3_N_4_ reproduced with permission from [21]. Copyright 2018, Elsevier; (**c**) V2*p*; (**d**) the percentage of (N_b_ + Nc) as a function of X in V-pg-C_3_N_4_-X.

To further reveal the influence of protonation on the surface structure of V-pg-C_3_N_4_, we performed N1*s* and V2*p* X-ray photoelectron spectroscopy (XPS) analysis for V-g-C_3_N_4_ and V-pg-C_3_N_4_-0.46. As shown in Figure 2a, the high-resolution spectra of N1*s* can be deconvoluted into three peaks. The major peak with a binding energy of 398.4 eV is ascribed to triazine nitrogen (C-N=C, N_a_ in Figure 2b) [40]. The second peak at 399.3 eV corresponds to sp^3^-hybridized three-coordinate N species (N-(C)_3_ and C-N(-C)-H, N_b_ in Figure 2b) [41,42], and the peak with the highest binding energy can be assigned to sp^3^-hybridized surface amino groups (e.g., -NH_2_ and -NH-, N_c_ in Figure 2b) at the edge of g-C_3_N_4_ sheets [21,43]. Interestingly, the relative intensities of these peaks are different for V-pg-C_3_N_4_-0.46 and V-g-C_3_N_4_, suggesting the different composition of N species. Specifically, the percentage of N_a_ decreases from 0.72 on V-g-C_3_N_4_ to 0.59 on V-pg-C_3_N_4_-0.46. This result is consistent with the reduced FT-IR band intensity at 1200–1650 cm^−1^, suggesting the presence of HCl in the preparation process indeed changes the surface heterocycle structure of C_3_N_4_. The percentage of N_b_ increases from 16% for V-g-C_3_N_4_ to 26% for V-pg-C_3_N_4_-0.46 while the percentage of N_c_ increases from 12% for V-g-C_3_N_4_ to 14% for V-pg-C_3_N_4_-0.46, suggesting the cleaved heterocycles were converted into groups with sp^3^-hybridized N species. The changed surface structure of g-C_3_N_4_ support eventually alters the surface state of supported vanadia. As shown in Figure 2c, the V2*p*_3/2_ spectra can be deconvoluted into two peaks at 515.3 and 517.1 eV, which are characteristic of V^4+^ and V^5+^ species, respectively [19]. Notably, the molar ratio of V^4+^/V^5+^ changes from 0.31/0.69 for V-g-C_3_N_4_ to 0.21/0.79 for V-pg-C_3_N_4_-0.46. This is likely due to the stronger interaction between vanadia and protonated g-C_3_N_4_ support in V-pg-C_3_N_4_-0.46 [22]. Specifically, the abundant N_b_ and N_c_ species in V-pg-C_3_N_4_-0.46 provide more anchoring sites for vanadia species, which improve the dispersion of vanadia and promote the electron transfer between vanadia and the g-C_3_N_4_ support.

To quantify the influence of HCl addition on the surface structure of V-pg-C_3_N_4_-X (X corresponds to mol HCl/mol N), we further investigated the XPS spectra for other V-pg-C_3_N_4_-X catalysts and plotted the percentage of (N_b_ + N_c_) as a function of X. As shown in Figure 2d, the percentage of (N_b_ + N_c_) exhibits a volcano plot versus X. Specifically, (N_b_ + N_c_)/N increases from 28% for V-g-C_3_N_4_ (X = 0) and 36% for V-pg-C_3_N_4_-0.09 (X = 0.09) to 41% for V-pg-C_3_N_4_-0.46 (X = 0.46), and then decreases to 31% for V-pg-C_3_N_4_-0.93 (X = 0.93). The maximum (N_b_ + N_c_)/N is achieved at X = 0.46, suggesting a proper amount of HCl addition is critical to the surface structure. When X is lower than 0.46, the amount of HCl is too small to change the bulk structure of g-C_3_N_4_ (Figure 1a). The increase in X would promote the hydrolysis of the aromatic CN heterocycles on the surface of g-C_3_N_4_ and therefore lead to increased (N_b_ + N_c_)/N, but when X is larger than 0.46, the excessive HCl not only hydrolyzes the aromatic CN heterocycles but also promotes the exfoliation of bulk g-C_3_N_4_. The further increase in X would expose more N_a_ on the surface and therefore decrease (N_b_ + N_c_)/N. As previously reported in the literature [3,44], the amino groups on the surface can react with VO(acac)_2_ to immobilize vanadia species. It is therefore expected that higher vanadia dispersion would be achieved by V-pg-C_3_N_4_ with larger (N_b_ + N_c_)/N. To confirm this hypothesis, we calculated the surface V/N molar ratio. Interestingly, it first increases from to 0.8% for V-pg-C_3_N_4_-0.05 to 2.1% for V-pg-C_3_N_4_-0.46 and then decreases to 1.2% for V-pg-C_3_N_4_-0.92. The similar trend of (N_b_ + N_c_)/N and V/N molar ratio confirms that high surface amino groups are beneficial for vanadia dispersion.

### 2.2. Microstructures of V-g-C_3_N_4_ and V-pg-C_3_N_4_

**Figure 3:**
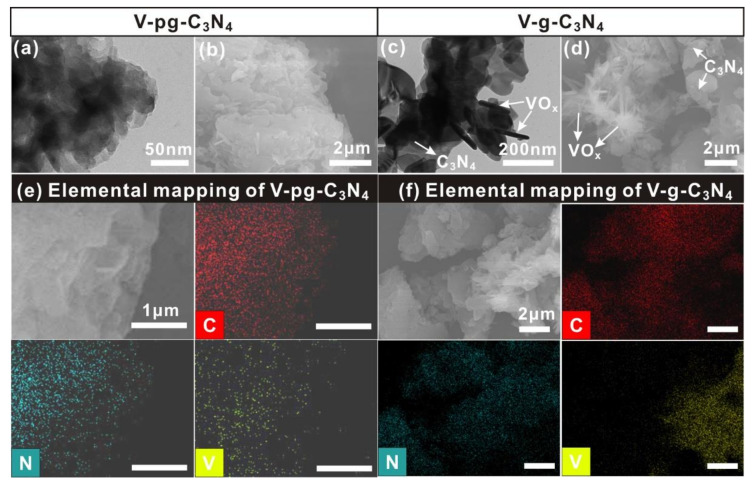
TEM (**a**) and SEM (**b**) images of V-pg-C_3_N_4_-0.46; TEM (**c**) and SEM (**d**) images of V-g-C_3_N_4_. Elemental mapping of V-pg-C_3_N_4_-0.46 (**e**) and (**f**) V-g-C_3_N_4_.

The different surface chemical states are associated with the different microstructures of V-g-C_3_N_4_ and V-pg-C_3_N_4_-0.46 (Figure 3). As shown in Figure 3a and 3b, V-pg-C_3_N_4_-0.46 presents a thin lamellar and platelet-like structure, which is similar to that of g-C_3_N_4_. Elemental mapping of V-pg-C_3_N_4_ shows a homogeneous distribution of C, N, and V (Figure 3e). No obvious aggregation of vanadia on the surface of g-C_3_N_4_ was observed, confirming the high dispersion of vanadia species over the support. In stark contrast, the transmission electron microscope (TEM) and scanning electron microscope (SEM) images of V-g-C_3_N_4_ exhibit two distinct characters (Figure 3c and 3d). The thin lamellar and platelet-like structures are characteristic of g-C_3_N_4_ while the nanorods are vanadia. Elemental mapping of V-g-C_3_N_4_ (Figure 3f) shows an inhomogeneous distribution of C, N, and V elements, which further confirms the phase separation observed by TEM and SEM. The distinct microstructures between V-g-C_3_N_4_ and V-pg-C_3_N_4_-0.46 likely originate from the different surface structure and interfacial interaction. According to our previous publications, strong interfacial interaction benefits the high dispersion of precursors on the support surface, eventually leading to the high dispersion of supported nanomaterials. For example, strong electronic interaction between Bi^3+^ and ZnO (or TiO_2_) motivates high dispersion of Bi precursors on ZnO (or TiO_2_) [45]. During the following transformation of Bi^3+^ into BiOI or Bi_2_O_3_, the highly dispersed Bi^3+^ favors bounded nucleation and growth, which eventually lead to the high dispersion of BiOI or Bi_2_O_3_ on the supports [46,47]. For g-C_3_N_4_, the interaction between amino groups and vanadium species is significantly higher than that between triazine nitrogen and vanadium species [4]. When HCl is added to the preparation of V-pg-C_3_N_4_-0.46, the surface of g-C_3_N_4_ is protonated and can provide more amino groups to anchor vanadium species. The bounded vanadium precursors in situ convert into vanadia and therefore achieve high dispersion on the surface of protonated g-C_3_N_4_. In the absence of HCl, the surface amino groups are limited and the interaction between g-C_3_N_4_ and vanadium species is too weak to drive the high dispersion of vanadium precursors. The free nucleation and growth of vanadium species eventually produce vanadia nanorods that are separated from the g-C_3_N_4_ support. It is important to highlight that in traditional synthetic methods, the high dispersion of vanadia in V-g-C_3_N_4_ was usually achieved by increasing the surface area of g-C_3_N_4_ (e.g., exfoliation or mesopores) [19,21]. In this study, however, the high dispersion of vanadia in V-pg-C_3_N_4_ was achieved without changing the bulk structure of g-C_3_N_4_. The similar bulk structure, surface area but distinct vanadia dispersion between V-g-C_3_N_4_ and V-g-C_3_N_4_ suggest the decisive factor for high vanadia dispersion is the surface structure of g-C_3_N_4_ rather than the surface area.

### 2.3. Catalytic Performance of V-pg-C_3_N_4_

**Figure 4:**
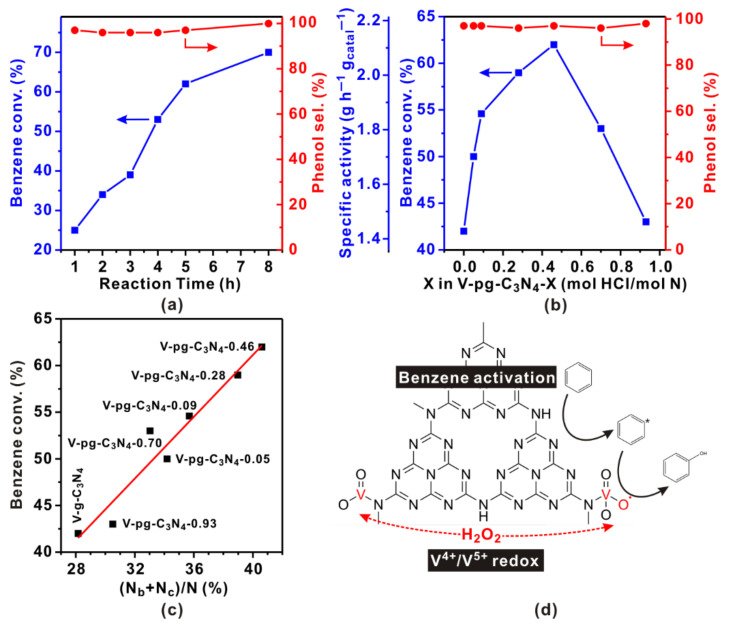
(**a**) Catalytic activity as a function of reaction time over V-pg-C_3_N_4_-0.46; (**b**) Catalytic activity (5 h) as a function of the HCl amount used in the preparation of V-pg-C_3_N_4_-X. Reaction conditions: benzene (1.0 mL, 11.3 mmol), hydrogen peroxide (30 wt%, 3.0 mL, 29.6 mmol), acetonitrile (4.8 mL), acetic acid (1.2 mL), catalyst (0.06 g, 0.022 mmol V), T = 333 K. (**c**) Benzene conversion as a function of (N_b_ + N_c_)/N over V-pg-C_3_N_4_-X. (**d**) A possible reaction mechanism for the direct hydroxylation of benzene to phenol over V-pg-C_3_N_4_ catalysts.

The catalytic performance of the catalysts was tested in direct hydroxylation of benzene to phenol using H_2_O_2_ as the oxidant. According to the literature, H_2_O_2_ was relatively stable in weak acidity conditions [48]. To this end, a mixture of acetonitrile and acetic acid was used as a solvent. Control experiment suggests that both g-C_3_N_4_ and protonated g-C_3_N_4_ display no activity in the absence of vanadia species, which is associated with its poor activity in H_2_O_2_ activation [4]. Interestingly, once vanadia was supported, V-g-C_3_N_4_ exhibited considerable conversion of benzene, suggesting vanadia species can activate H_2_O_2_ to hydroxylate benzene. Before evaluating the catalytic performances of different vanadia catalysts, we first optimized the reaction conditions. The optimal reaction temperature is 333 K as it achieves high phenol yield and avoids the volatilization of benzene (boiling point of 353 K). Figure 4a plots benzene conversion and phenol selectivity as a function of reaction time over V-pg-C_3_N_4_-0.46. The selectivity of phenol is always higher than 95%, which is similar to that for other reported C_3_N_4_-based catalysts (Table 1). According to the literature [4], the high selectivity of phenol is closely related to the unique structure of C_3_N_4_. Xu et al. [19] carried out benzene temperature-programmed desorption experiments over g-C_3_N_4_, V_2_O_5_, and V/g-C_3_N_4_ and found that benzene preferred to adsorb on g-C_3_N_4_ instead of vanadia. Notably, g-C_3_N_4_ features aromatic s-triazine rings and π electrons, which facilitate a strong planar adsorption of benzene on the surface of g-C_3_N_4_. However, once the adsorbed benzene is hydroxylated to phenol by H_2_O_2_, the aromatic ring alters the symmetry of the molecular orbital [17]. The resulting non-planer adsorption of phenol is much weaker than the planer adsorption of benzene. As a result, phenol tends to desorb from g-C_3_N_4_. The conversion of benzene rapidly increases from 25% to 62% along with the reaction time extension from 1 h to 5 h. Further increasing the reaction to 8 h, the conversion slightly increases to ~70%, likely due to the runout of H_2_O_2_. To this end, the products of 5-h reaction were used to evaluate the catalytic performance of different catalysts.

Figure 4b plots the catalytic performance of V-pg-C_3_N_4_-X as a function of X, i.e., the amount of HCl (mol HCl/mol N) used in the preparation of V-pg-C_3_N_4_-X. When X = 0, it is also called V-g-C_3_N_4_. Interestingly, all V-pg-C_3_N_4_ catalysts exhibit higher benzene conversion than V-g-C_3_N_4_, suggesting that the addition of HCl in the preparation benefits the catalytic performance. In particular, the benzene conversion over V-pg-C_3_N_4_-0.46 is 62%, which is significantly higher than that of V-g-C_3_N_4_ (42%). Considering that these two catalysts have similar XRD patterns, surface area, and vanadia loading amount, the mass transfer performances should be the same. Their different catalytic performances most likely originate from their distinct surface structures, especially their different amino contents and vanadia dispersion which influence the activation of H_2_O_2_. Second, the catalytic performance of V-pg-C_3_N_4_-X exhibits a volcano plot versus X, suggesting the amount of HCl added in the preparation is critical. Interestingly, the plot follows the same trend as that of the volcano plot of (N_b_ + N_c_)/N versus X. Specifically, when X is lower than 0.46, (N_b_ + N_c_)/N and benzene conversion increase along with the increase in X, but when X exceeds 0.46, they decrease. A maximum of 62% benzene conversion with 60.1% phenol yield is achieved at 333 K over V-pg-C_3_N_4_-0.46, a catalyst with the highest (N_b_ + N_c_)/N. The mass specific activity of V-pg-C_3_N_4_-0.46 is as high as 2.11 g g_cat_^−1^ h^−1^, which exceeds most reported results (Table 1). Moreover, when we plot benzene conversion as a function of the percentage of (N_b_ + N_c_), a quasi-linear relationship is obtained (Figure 4c). These results are consistent with the hypothesis that high vanadia dispersion enabled by abundant surface amino groups benefits the hydroxylation of benzene to phenol over V-pg-C_3_N_4_.

### 2.4. Reaction Mechanism of Benzene Hydroxylation over V-pg-C_3_N_4_

According to the literature [18,19,49], the hydroxylation of benzene to phenol over V-pg-C_3_N_4_ follows a synergistic mechanism (Figure 4d). Specifically, benzene is adsorbed and activated on the g-C_3_N_4_ support while H_2_O_2_ is involved in the V^4+^/V^5+^ redox cycle. Considering that phenol is produced by the reaction between activated benzene and V^5+^–O–O^•^, the overall reaction rate of benzene hydroxylation depends on both activation reactions. In general, due to the abundant tri-s-triazine moieties and the facile electron transfer from C_3_N_4_ to benzene, the adsorption and activation of benzene over g-C_3_N_4_ can easily occur. In contrast, the oxidation of V^4+^ into V^5+^–O–O^•^ by H_2_O_2_ may be limited by the number of active vanadia sites. In this study, the surface structure (amino groups and vanadia dispersion) of V-pg-C_3_N_4_ was regulated without changing the bulk structures, providing an ideal model to investigate the influence of surface structure on the catalytic performance of benzene hydroxylation. According to the above discussion, the addition of HCl in the preparation of V-pg-C_3_N_4_-X leads to the protonation of g-C_3_N_4_. The resultant increase in surface amino groups provides more anchoring sites for vanadia species and therefore achieves higher vanadia dispersion. Considering that V-pg-C_3_N_4_-0.46 has a similar surface area and vanadia loading amount as V-g-C_3_N_4_, the higher vanadia dispersion means it has more active vanadia sites for H_2_O_2_ activation, which therefore leads to superior catalytic performance in benzene hydroxylation to phenol.

## 3. Materials and Methods

### 3.1. Preparation of g-C_3_N_4_

g-C_3_N_4_ was synthesized by the direct pyrolysis of melamine. Briefly, 5.0 g of melamine was placed in a crucible with a cover and then heated at 550 °C for 2 h with a ramping rate of 5 °C min^−1^.

### 3.2. Preparation of V-pg-C_3_N_4_

Typically, 0.3 g of as-synthesized g-C_3_N_4_ was dispersed into 10 mL of water containing 0.032 g of vanadyl acetylacetonate. After vigorous stirring for 20 min, a certain amount of HCl (ca. 37 wt%) was added to the above mixture dropwise. The mixture was stirred at room temperature for 2 h and subsequently heated at ~80 °C until the solvent was totally evaporated. The obtained product was calcined in the crucible with a cover at 300 °C for 2 h with a ramping rate of 2 °C min^−1^. The obtained sample was labeled as V-pg-C_3_N_4_-X, where X (X = 0.05, 0.09, 0.28, 0.46, 0.70, and 0.93) is the molar ratio between HCl and N atom in g-C_3_N_4_ (mol HCl/mol N). V-g-C_3_N_4_ was synthesized in the same way as V-pg-C_3_N_4_-X except that no HCl was added. Inductively coupled plasma mass spectrometry analysis confirmed that the mass loading of V was around 1.5 wt.%.

### 3.3. Characterizations

Scanning electron microscopy (SEM) images and elemental mapping were obtained using a Hitachi S4800 SEM microscopy equipped with an energy dispersive X-ray spectroscopy (EDS). Transmission electron microscopy investigations were performed on a HT7700 electron microscopy. X-ray diffraction patterns were collected using a Rigaku Ultimate IV diffractometer (Cu Kα radiation, 40 kV and 30 mA). Fourier transform infrared (FT-IR) spectra were recorded on a Thermo Nicolet 380 spectrometer. X-ray photoelectron spectroscopy (XPS) measurements were performed on a VG Scientific ESCALAB Mark II spectrometer.

### 3.4. Catalytic Performance Evaluation

The direct hydroxylation of benzene to phenol was performed in a three-neck round-bottom flask equipped with a reflux condenser. Typically, 1 mL of benzene, 4.8 mL of acetonitrile, 1.2 mL of 80 wt.% acetic acid, and 60 mg of catalyst were added to a 25 mL three-necked flask. The solution was stirred at 60 °C for 20 min to ensure adsorption equilibrium of benzene on the catalyst. Then, 3 mL of aqueous H_2_O_2_ solution (30 wt.%, 29.5 mmol) was dropwise added into the reactor within 2 min under vigorous stirring. The reaction was conducted at 60 °C for 1–8 h. After the reaction, the mixture was separated and the liquid products were analyzed by Shimadzu LC-20AD HPLC with an Ultimate XB-C18 column.

Conversion of benzene (*Conv*.) and selectivity to phenol (*Sel*.) were calculated as follows:

$$Conv.=\frac{n_{phenol}+n_{BQ}+n_{HQ}+n_{CA}}{n_{benzene}+n_{phenol}+n_{BQ}+n_{HQ}+n_{CA}}\times 100\%$$

$$Sel.=\frac{n_{phenol}}{n_{phenol}+n_{BQ}+n_{HQ}+n_{CA}}\times 100\%$$

where *n_benzene_*, *n_phenol_*, *n_BQ_*, *n_HQ_*, *n_CA_* are the molar amount (mol) of benzene, phenol, benzoquinone (*BQ*), hydroquinone (*HQ*), and catechol (*CA*).

The mass specific activity for each catalyst was calculated as follows:

$$Specific\;activity\;\left(g\;h^{-1}g_{catal.}^{-1}\right)=\frac{n_{phenol}\times M_{phenol}}{W_{catal.}\times t}$$

where M_phenol_, W_catal_., and t represent the formula weight (g mol^−1^) of phenol, the mass of overall catalyst (*g*), and the reaction time (*h*), respectively.

## 4. Conclusions

We developed a one-pot synthesis of V-pg-C_3_N_4_ which protonates g-C_3_N_4_ and loads vanadia simultaneously. Characterizations suggest that the protonation of g-C_3_N_4_ in diluted HCl solution facilitates the generation of amino groups (NH/NH_2_) on the surface without changing its bulk structure. The generated amino groups serve as anchoring sites to stabilize the highly dispersed vanadia species, which efficiently activate H_2_O_2_ into radical-containing V^5+^ species to react with the adjacent benzene activated over g-C_3_N_4_. Interestingly, the percentage of surface amino groups and the vanadia dispersion can be easily regulated by the amount of HCl added in the preparation. The optimal catalyst, V-pg-C_3_N_4_-0.46, achieves 60% benzene conversion and 99.7% phenol selectivity at 60 ^o^C with H_2_O_2_ as the oxidant. The mass-specific activity of V-pg-C_3_N_4_-0.46 is as high as 2.11 g g_cat_^−1^ h^−1^, which exceeds most reported results.

## Figures and Tables

**Table 1:** The catalytic activity of various vanadia- and g-C_3_N_4_-based catalysts for benzene hydroxylation.

Catalyst	V_benzene_ (mL)	V_H_2_O_2__ (mL)	W_catal_. (mg)	T (K)	t (h)	Conv. (%)	Sel. (%)	Specific Activity (h^−1^) ^a^	Ref.
0.4V-g-C_3_N_4_	1.0	3	60	333	6	17.7	100	0.52	[4]
H_5_PMo_10_V_2_O_40_/pg-C_3_N_4_	1.0	4.1	100	333	8	25.8	99.7	0.34	[22]
VO/MCM-41-NH_2_	0.9	1	100	333	1	58.6	18.5	1.03	[6]
8V-g-C_3_N_4_	1.0	3.5	40	343	4	24.6	99.2	1.61	[18]
Fe-g-C_3_N_4_	1	3	50	333	2	17.5	99.0	1.84	[23]
V_2_O_5_-mp-C_3_N_4_	1.5	3	60	333	3	18.7	95.9	1.59	[19]
VO-peg-C_3_N_4_	1.0	3	75	333	4	11.7	97.9	0.40	[21]
g-C_3_N_4_ QD/Fe-SBA-15	3	6	120	333	3	41,7	98.8	3.01	[24]
VO_x_-SBA-16	0.3	1.5	10	333	4	13.8	97.5	1.06	[25]
HPMoV/NH_2_-SBA-15	1.0	3.0	100	333	6	20.0	95.0	0.33	[26]
VO*_x_*-TiO_2_	2.6	6	180	333	5	26.3	90.0	0.72	[27]
PMoV_2_/SiO_2_	1.0	3.0	150	333	6	21.6	100	0.25	[28]
V-C-600	0.4	1.4	20	343	3	31.8	94.9	2.12	[29]
VO*_x_*-GO	1.0	3.5	40	338	3	23.1	98.4	1.99	[30]
Ce_0.07_-0.07 V-g-C_3_N_4_	1.0	3.5	40	343	4	33.7	95.9	2.13	[8]
V-pg-C_3_N_4_-0.46	1.0	3.0	60	333	5	62.0	97.0	2.11	p.w. ^b^
V-pg-C_3_N_4_-0.46	1.0	3.0	60	333	1	25.0	97.0	4.26	

^a^ Specific activity is calculated based on the mass of synthesized phenol per gram of whole catalyst per hour. ^b^ Present work.

## Data Availability

The data presented in this study are available on request from the corresponding author.

## Sample Availability

Samples of the compounds V-g-C_3_N_4_ and V-pg-C_3_N_4_ are available from the authors.
